# Achieving High Strength and Low Yield Ratio via Direct Quenching and Aging in Cu-Precipitation-Strengthened Steel

**DOI:** 10.3390/nano16010066

**Published:** 2026-01-02

**Authors:** Xinghao Wei, Youjing Zhang, Yajie Wen, Chaofei Yang, Xinghua Wang, Jiajia Niu, Renfu Wang

**Affiliations:** National Key Laboratory of Marine Corrosion and Protection, Luoyang Ship Material Research Institute, Luoyang 471023, China; weixinghao163@163.com (X.W.);

**Keywords:** Cu-precipitation-strengthened steel, high strength, low yield ratio, precipitation strengthening, strengthening and toughness mechanisms

## Abstract

The high yield ratio remains a critical challenge restricting the widespread application of ultra-high-strength steels. This study investigates a direct quenching and aging (DQA) route without solution treatment in a Cu-precipitation-strengthened steel, aiming to achieve high strength combined with a low yield ratio, and compares it with the conventional solution treatment plus aging (SQA) process. The DQA sample exhibits an excellent yield strength of 1205 MPa, a low yield ratio of 0.93, and an impact energy of 105 J at −20 °C. Microstructural analysis reveals that the high dislocation density and refined grain structure generated during rolling provided numerous nucleation sites for fine, dense Cu precipitates during DQA treatment, thereby enhancing precipitation strengthening. The reduced yield ratio is primarily attributed to the high initial dislocation density and deformation substructure, which enhance work-hardening capacity and consequently lower the yield ratio. The toughness mechanisms of both processes are also discussed in detail. These findings offer valuable insights into optimizing the strength–toughness balance of ultra-high-strength steels.

## 1. Introduction

High-strength structural steel is an indispensable material in modern industry, with critical applications in heavy machinery, shipbuilding, and offshore platforms [1,2,3]. These applications demand not only ultra-high-strength to reduce weight and increase load-bearing capacity but also sufficient toughness to ensure structural integrity and safety under extreme conditions such as low temperature and dynamic loads. Moreover, good weldability is essential to lower manufacturing costs [4,5,6].

Precipitation strengthening is a promising approach for achieving high strength in such steels. Among these, Cu-precipitation-strengthened steels are particularly attractive, as they can substitute for carbon strengthening while improving strength and weldability without compromising toughness; representative examples include HSLA-100 and HSLA-115 steels [7,8]. Adding 2–4 wt.% Cu to low-carbon steel enables the formation of Cu precipitates during aging, which is the core mechanism for strength enhancement without severe hot cracking [9]. However, as strength increases, precipitation strengthening primarily enhances yield strength but contributes little to tensile strength. In ultra-high-strength low-carbon steels, a greater reliance on precipitation strengthening generally leads to an increased yield ratio, in some cases exceeding 99% [10,11]. A high yield ratio compromises crash resistance and overload capacity, thereby limiting the application of high-strength precipitation-strengthened steels [12,13].

Online direct quenching and tempering, as part of the thermomechanical controlled process (TMCP), have been widely adopted in low-alloy steels. Compared with conventional offline reheat quenching and tempering, this process avoids repeated austenitization after rolling, thereby preserving the deformation-induced dislocations from rolling in the non-recrystallized austenite region, producing a refined quenched microstructure with high dislocation density [14,15]. This significantly enhances work hardening and leads to a reduced yield ratio [16,17]. However, some studies show that in precipitation-strengthened steel, direct quenching can promote the homogeneous nucleation of precipitates, reduce the coarsening rate of precipitates, and improve the effect of precipitation strengthening [18]. The interaction between TMCP parameters (e.g., cooling rate, finishing temperature) and Cu precipitation kinetics is complex, and unoptimized processes may still result in uneven precipitate distribution or compromised toughness [19]. Fu et al. found that in CuNiAl steels, the yield strength ratio of direct-quenched specimens still approaches 99% after aging, accompanied by a notable loss of toughness [20]. Thus, the strengthening and toughening mechanisms of precipitation-strengthened steel subjected to direct quenching remain insufficiently understood, warranting further investigation.

In this study, we applied direct quenching and aging (DQA) in Cu-precipitation-strengthened steel and systematically compared the results with those of conventional solution treatment and aging (SQA). The aim was to elucidate the influence of these two processing routes on microstructural evolution and mechanical properties, with particular emphasis on balancing strength and toughness as well as reducing the yield ratio. Our findings demonstrate that DQA provides an effective alternative to conventional processing and offers a viable strategy for developing high-strength steels that meet the stringent demands of modern engineering applications.

## 2. Materials and Methods

**Figure 1:**
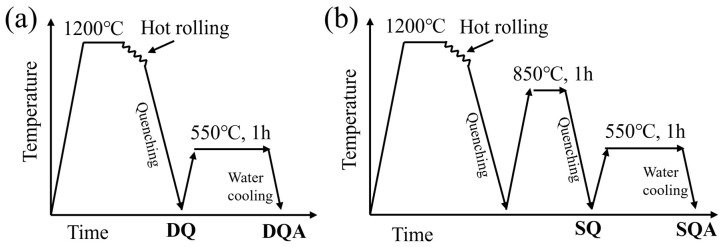
The schematic of the manufacturing routes. (**a**) DQ and DQA samples, (**b**) SQ and SQA samples.

The Cu-precipitation-strengthened steel was smelted in a 50 kg vacuum induction furnace, forged into 100 mm × 120 mm × 90 mm square ingots, and air-cooled to room temperature. The forged billets were heated to 1200 °C, hot-rolled into 30 mm thick plate through rough rolling and finish rolling and then quenched to room temperature. The sample was designated as DQ (direct quenching). The DQ sample was subsequently solution-treated at 850 °C for 1 h and then water quenched, hereafter referred to as SQ (solution and quenching). Both the DQ and SQ samples were aged at 550 °C for 1 h, followed by water cooling, and are denoted as DQA and SQA, respectively, as shown in Figure 1. The chemical composition of the steel was determined in accordance with ASTM E415 and ASTM E1019, listed in Table 1. The carbon content was measured following the combustion-infrared absorption method specified in ASTM E1019.

Tensile tests were conducted on an INSTRON 5565 testing machine at a strain rate of 0.001/s in the transverse direction. Three specimens were tested for each condition, and the average values with standard deviations are reported. Yield strength was defined using the 0.2% offset method.

Charpy V-notch (CVN) impact tests were carried out on a Zwick/Roell 450 J instrumented impact machine at −80 °C in the transverse direction. Standard CVN specimens (55 mm × 10 mm × 10 mm) with V-notches introduced along the thickness direction by wire electrical discharge machining were prepared in accordance with ASTM E23-18. Three specimens were tested for each condition, and the results are presented as averages with associated error ranges.

Microstructural characterization was performed using an OLYMPUS GX71 optical microscope (OM). Prior austenite grain boundaries were revealed by picral etching, while general microstructures were etched with 4% nital solution. X-ray diffraction (XRD) was performed on a PANalytical X’Pert Pro diffractometer using Cu Kα radiation. Electron backscatter diffraction (EBSD) was carried out on an FEI Scios 2 dual-beam scanning electron microscope (SEM). Transmission electron microscopy (TEM) observations were performed on a Thermo Scientific Talos F200X microscope operating at 200 kV. High-angle annular dark-field (HAADF) imaging and energy-dispersive X-ray spectroscopy (EDS) were conducted at an accelerating voltage of 300 kV. The TEM samples were prepared via an ion beam thinning procedure.

## 3. Results

Table 2 summarizes the yield strength, tensile strength, yield ratio, elongation, and impact energy at −20 °C (KV_2_ −20 °C) of the DQ, DQA, SQ, and SQA specimens. The yield strength of SQ sample decreased by ~80 MPa compared with the DQ sample, while the impact energy increased by 14 J. Compared with the DQA specimen, the SQA sample exhibited a 112 MPa reduction in yield strength, a 187 MPa reduction in tensile strength, a 0.06 increase in yield ratio, and a 73 J improvement in impact toughness.

**Figure 2:**
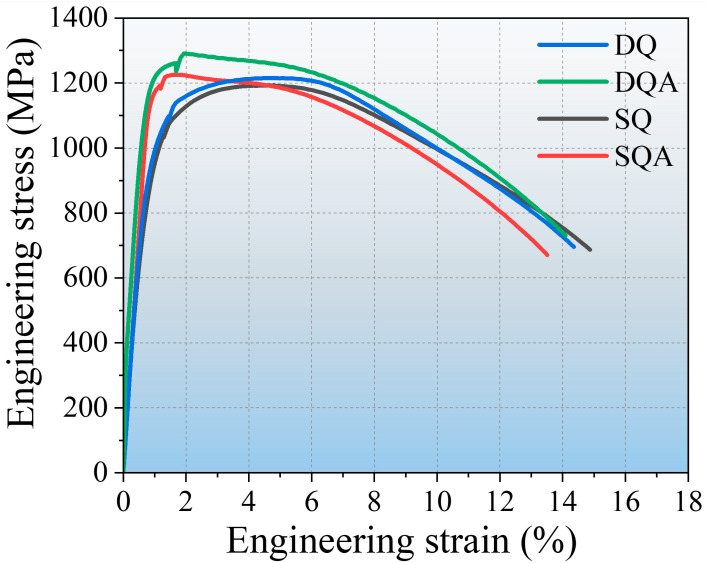
Engineering tensile stress–strain curves of the experimental steel.

Figure 2 presents the engineering tensile stress–strain curves of the experimental steel. The SQA samples rapidly reached their tensile strength after yielding, followed by a sharp decline; the difference between yield and tensile strength was only 11 MPa. In contrast, the DQA specimens continued to work harden after yielding, with strength increasing by 86 MPa before reaching the ultimate tensile strength.

**Figure 3:**
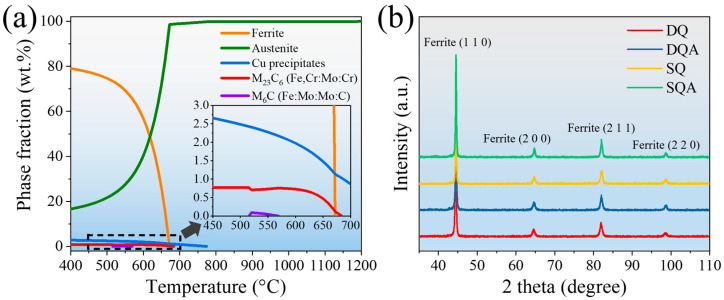
(**a**) Thermodynamic calculation showing the phase fraction as a function of temperature, (**b**) XRD patterns of the DQ, DQA, SQ, and SQA specimens.

Figure 3a presents the thermodynamic equilibrium phase diagram of the experimental steel [21]. This phase diagram was obtained through Thermo-Calc (2023a) software, with the TCFE11 database. At 550 °C, the equilibrium fractions of Cu precipitates, M_23_C_6_, and M_6_C were ~2.33%, 0.74%, and trace amounts, respectively. The XRD patterns of the DQ, DQA, SQ, and SQA specimens (Figure 3b) revealed a single BCC structure, with no detectable austenite or precipitates.

**Figure 4:**
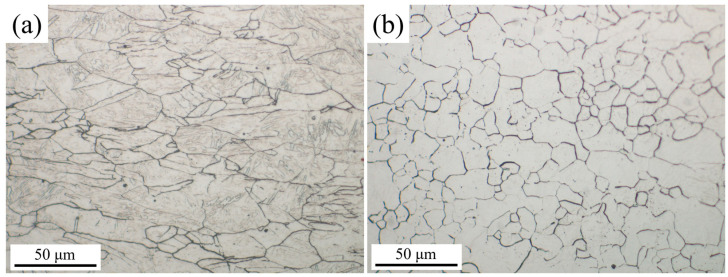
Prior austenite grain boundary micrographs of (**a**) DQ and (**b**) SQ samples.

To investigate the influence of processing on the matrix structure, the prior austenite grain boundaries of the DQ and SQ samples were examined (Figure 4). The DQ (Figure 4a) sample exhibits elongated polygonal austenite grains primarily due to the thermomechanical control processing (TMCP) characteristics of direct quenching. During rolling, the steel is deformed in the non-recrystallized austenite region, where deformation-induced dislocations accumulate and grain boundaries are stretched along the rolling direction. Immediately after rolling, direct quenching rapidly cools the steel to below the martensite start (M_S_) temperature. This rapid cooling inhibits static recrystallization and grain growth of austenite, preserving the deformed, elongated grain morphology formed during rolling. Additionally, the presence of fine subgrains within the elongated grains is attributed to the dislocation rearrangement under deformation, which is retained due to the suppressed recrystallization.

In contrast, the SQ (Figure 4b) sample displays equiaxed austenite grains with a fully recrystallized structure. The solution treatment involves heating the steel to a temperature above the austenitization temperature (A_C3_) and holding it for a sufficient duration. This process completely dissolves precipitates and eliminates deformation-induced microstructural features (e.g., dislocations, stretched grain boundaries) from the prior rolling stage. During holding, austenite undergoes full static recrystallization, where new equiaxed grains nucleate and grow uniformly to minimize the system’s free energy. Subsequent quenching fixes this recrystallized equiaxed grain morphology, resulting in the homogeneous, equiaxed structure observed in Figure 4b.

**Figure 5:**
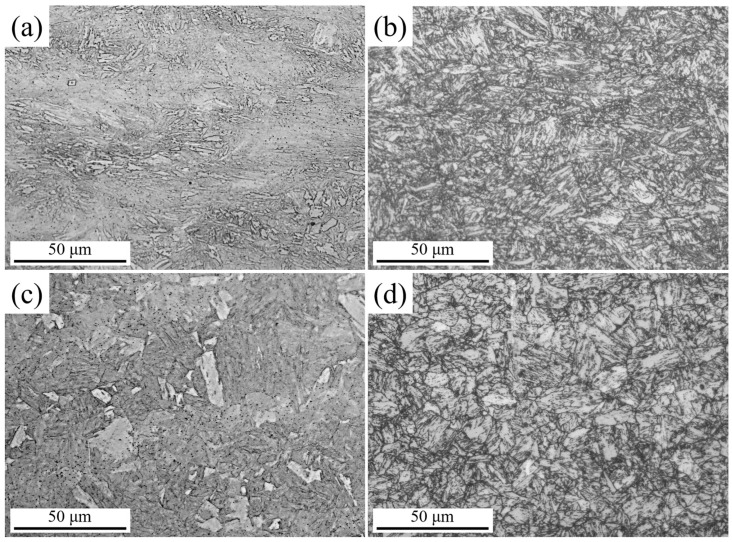
Metallographic images of (**a**) DQ, (**b**) DQA, (**c**) SQ, and (**d**) SQA specimens.

Figure 5 shows the metallographic images of DQ, DQA, SQ, and SQA specimens. The DQ sample mainly consisted of martensite with a high fraction of fine grains, whereas the DQA exhibited tempered martensite with similar fine grains. The SQ sample contained typical lath martensite, while the SQA sample contained tempered lath martensite.

**Figure 6:**
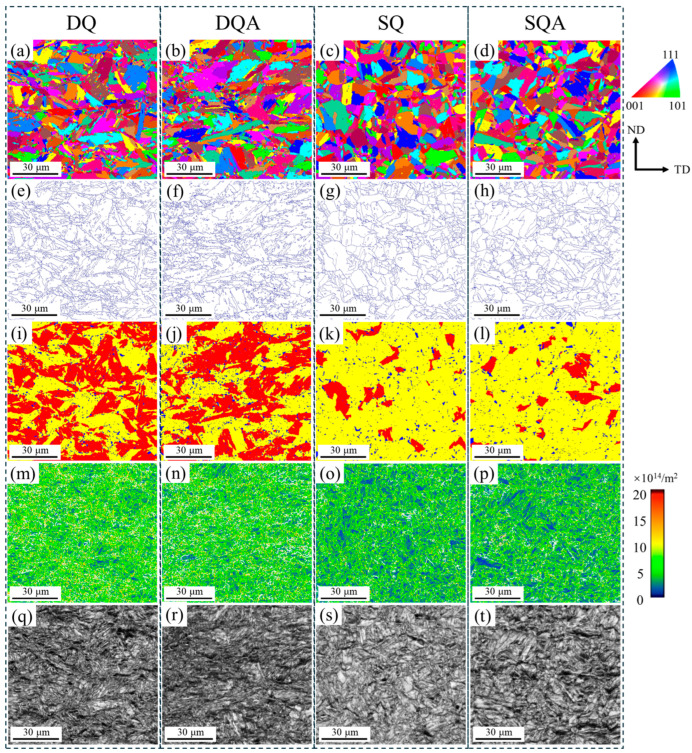
EBSD characterization results and SEM micrographs of DQ, DQA, SQ, and SQA samples: (**a**–**d**) Inverse Pole Figure (IPF) orientation maps showing the overall grain orientation distribution; (**e**–**h**) High-Angle Grain Boundary (HAGB) maps (blue lines represent HAGBs, ≥15°) indicating packet and block boundaries that act as crack propagation barriers; (**i**–**l**) Grain Orientation Spread (GOS) maps (color coding: blue = recrystallized grains, GOS < 1°; yellow = recovered grains, 1–5°; red = deformed grains, GOS > 5°); (**m**–**p**) Geometrically Necessary Dislocation (GND) density maps (warmer colors correspond to higher GND density) with calculated $\rho_{GND}$ values: (**m**) 8.62 × 10^14^ m^−2^ (DQ), (**n**) 8.22 × 10^14^ m^−2^ (DQA), (**o**) 5.22 × 10^14^ m^−2^ (SQ), (**p**) 4.97 × 10^14^ m^−2^ (SQA); (**q**–**t**) Corresponding SEM micrographs showing macroscopic microstructural features consistent with EBSD characterization regions.

EBSD and TEM analyses were performed on all conditions (Figure 6). The transformation of prior austenite grains into lath martensite produced several packets, each subdivided into blocks, which were further divided by laths. Packet and block boundaries correspond to high-angle grain boundaries (HAGBs, shown in blue in Figure 6e–f), which are effective barriers to crack propagation. The effective grain sizes (EGSs) of the DQ, DQA, SQ, and SQA specimens were 2.17 ± 0.12 μm, 1.89 ± 0.15 μm, 2.58 ± 0.11 μm, and 2.48 ± 0.14 μm, respectively.

Figure 6i–l present the grain orientation spread (GOS) map of DQ, DQA, SQ, and SQA samples. Blue regions (GOS < 1) correspond to recrystallized grains, yellow regions (1–5) to recovered grains, and red regions (>5) to deformed grains [22]. The fractions of recrystallized grains were 4%, 6%, 4%, and 4% for the DQ, DQA, SQ, and SQA samples, respectively. The corresponding fractions of recovered grains were 45%, 47%, 87%, and 87%, while those of deformed grains were 51%, 47%, 9%, and 9%. DQ and DQA samples retained a large fraction of deformed microstructures, whereas most deformed regions in SQ and SQA samples recovered during austenitization.

Figure 6m–p shows the geometrically necessary dislocations (GNDs) densities of the four DQ, DQA, SQ, and SQA specimens, with $\rho_{GND}$ values of 8.62 × 10^14^ m^−2^, 8.22 × 10^14^ m^−2^, 5.22 × 10^14^ m^−2^, and 4.97 × 10^14^ m^−2^, respectively. The dislocation densities of DQ and DQA samples were significantly higher than those of SQ and SQA samples.

**Figure 7:**
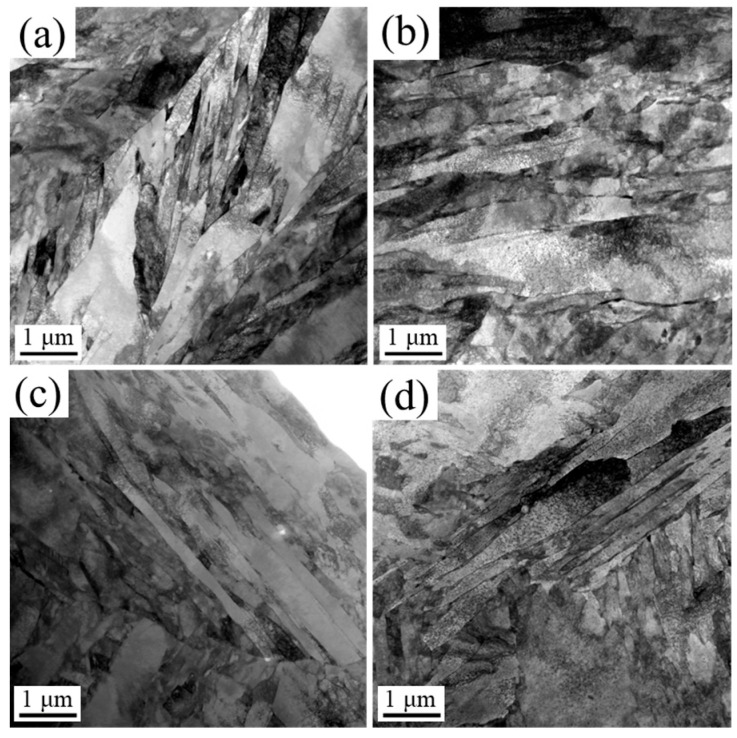
TEM micrographs of (**a**) DQ, (**b**) DQA, (**c**) SQ, and (**d**) SQA samples.

Figure 7 shows the bright-field TEM micrographs of DQ, DQA, SQ, and SQA samples. All samples exhibited lath martensitic microstructures. However, the laths in DQ and DQA samples were shorter and less uniform, owing to the presence of numerous fine grains, compared with those in SQ and SQA samples.

## 4. Discussion

### 4.1. Strengthening Mechanism

The yield strength of DQA sample is approximately 110 MPa higher than that of the SQA sample, while its yield ratio is lower. This improvement is primarily attributed to differences in grain size, dislocation density, and Cu precipitates [1].

**Figure 8:**
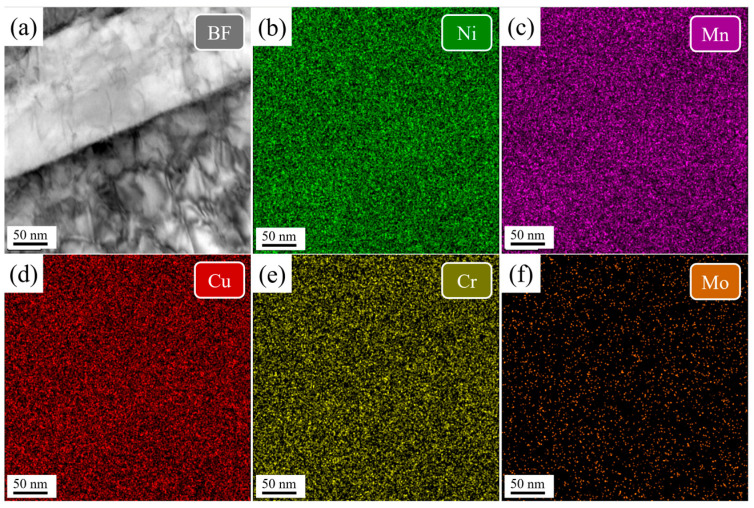
The TEM-EDS mapping of the martensitic lath in the DQ sample. (**a**) bright field image of the lath boundary, (**b**–**f**) element mapping.

**Figure 9:**
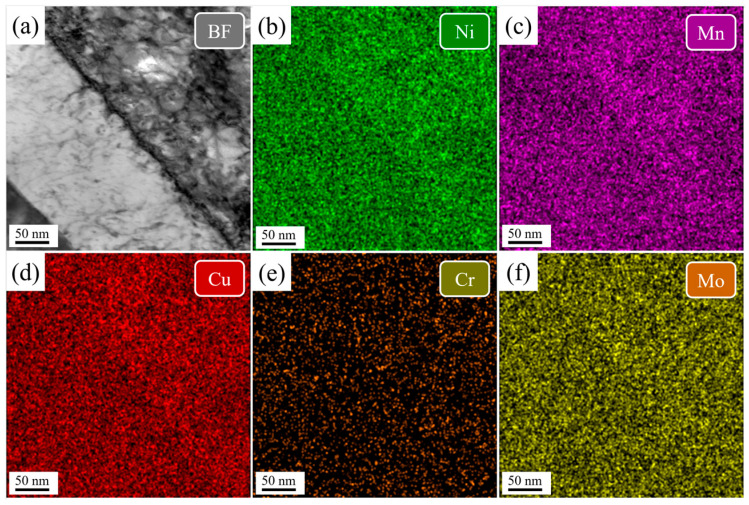
The TEM-EDS mapping of the martensitic lath in the SQ sample. (**a**) bright field image of the lath boundary, (**b**–**f**) element mapping.

Figure 8 and Figure 9 show TEM-EDS mapping of the martensitic lath in the DQ and SQ samples, respectively. In both cases, elements are uniformly distributed without evidence of segregation or precipitation.

**Figure 10:**
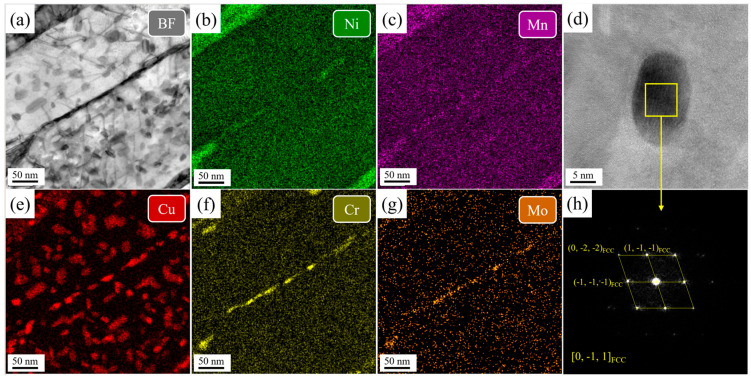
The TEM-EDS mapping of the martensitic lath and HRTEM image of Cu precipitates in the SQA sample. (**a**) bright field image of the lath boundary, (**b**,**c**,**e**,**f**,**g**) element mapping, (**d**) HRTEM image of Cu precipitates, (**h**) diffraction pattern of the Cu precipitate.

Figure 10 presents TEM-EDS elemental mapping and an HRTEM image of Cu-rich precipitates in the SQA sample. A high density of Cu precipitates, with an average diameter of 8.54 ± 2.3 nm, radius distribution of 3.2–9.5 nm, and a volume fraction of 1.9%, is observed. Fast Fourier transform (FFT) patterns confirm that these precipitates have an FCC structure. In addition, Cr and Mo segregate strongly to the lath boundaries, with minor segregation of Ni and Mn. Significant amounts of reversed austenite, enriched in Ni and Mn, are also detected both along and within the lath structure.

**Figure 11:**
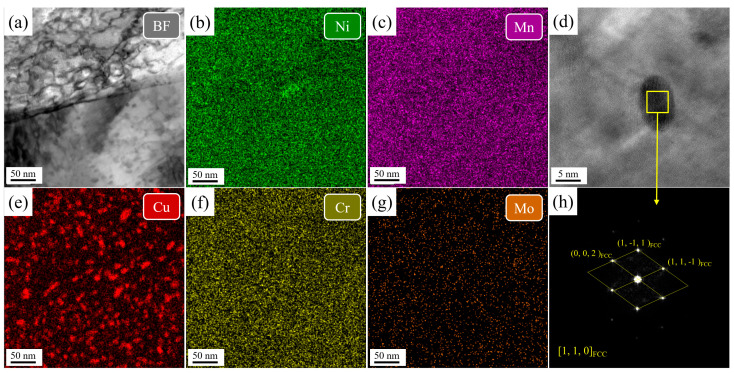
The TEM-EDS mapping of the martensitic lath and HRTEM image of Cu precipitates in the DQA sample. (**a**) bright field image of the lath boundary, (**b**,**c**,**e**,**f**,**g**) element mapping, (**d**) HRTEM image of Cu precipitates, (**h**) diffraction pattern of the Cu precipitate.

In contrast, Figure 11 shows that the DQA sample contains Cu precipitates with much higher number density and smaller size, averaging 5.7 ± 1.2 nm in diameter, radius distribution of 2.1–7.4 nm, and a volume fraction of 2.3%. FFT patterns indicate that these small-sized Cu precipitates also possess FCC structures. The high dislocation density and abundant deformation substructures in DQ provide abundant nucleation sites, accounting for the finer and denser Cu precipitates. Moreover, unlike in the SQA sample, no element segregation is detected at the lath boundaries of the DQA sample, because there is no solution treatment, and Cr, Mo and other elements are not evenly distributed in the structure. As a result, during aging, alloying elements are unable to sufficiently segregate to lath boundaries.

To quantify the strengthening mechanisms of DQA and SQA specimens, the yield strength was calculated by considering contributions from grain-boundary strengthening, solid-solution strengthening, dislocation strengthening, and precipitation strengthening. The overall yield strength σ can be expressed as [1,23,24,25,26]:

(1) $$\sigma ={\sigma_{GB}+\sigma }_{ss}+\sigma_{dislo}+\sigma_{ppt}$$

The contributions of grain boundary strengthening, dislocation strengthening, and solid-solution strengthening are given by [27,28,29]:

(2) $$\sigma_{GB}=\sigma_{0}+k_{y}d^{-\frac{1}{2}}$$

(3) $$\sigma_{dislo}=M\alpha Gb\sqrt{\rho }$$

(4) $$\sigma_{SS}=4570\left[C\right]+37\left[Mn\right]+83[Si]+38[Cu]+11[Mo]-30[Cr]$$

where $\sigma_{0}=50$ MPa is the friction stress [24,30], $k_{y}$ is the Hall-Petch constant for martensite packets (200 MPa μm^1/2^) [23], α = 0.2 is a constant, *M* is the Taylor factor (*M* = 3 for BCC metals), *G* is the shear modulus (*G* = 80 GPa for the α-Fe matrix), *ρ* represents the density of geometrically necessary dislocations (GNDs), b is the Burgers vector (b = 0.25 nm); and [Xᵢ] represents the solute concentration (wt.%).

The strengthening effect of FCC structure Cu precipitates was evaluated using the Orowan dislocation bypassing model [26,31,32]:

(5) $$∆\sigma_{Orowan}=0.1Gb\frac{f^{1/2}}{R}\ln\frac{R}{b}$$

where *f* is the volume fraction and *R* the mean radius of the precipitates. The Orowan strengthening contributions were estimated as ~259 MPa for the DQA sample and ~183 MPa for the SQA sample. Carbides also contributed via Orowan strengthening, providing increments of ~93 MPa (DQA) and ~149 MPa (SQA) [23,33,34].

**Figure 12:**
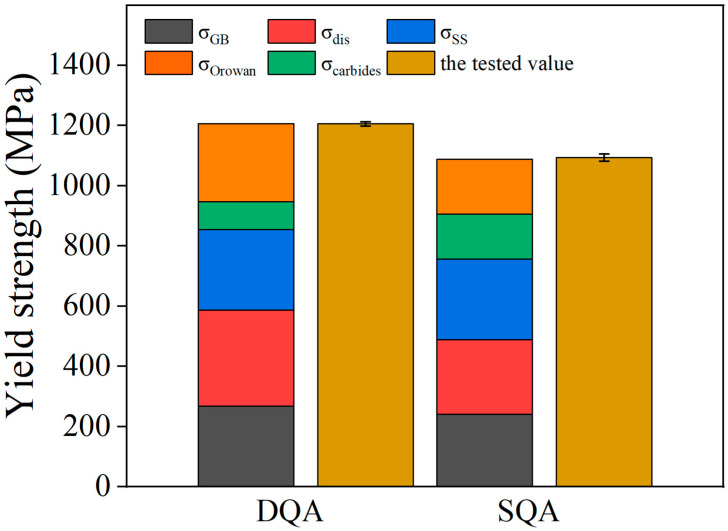
Theoretical and experimental values of yield strength for DQA and SQA specimens, as well as calculations of various strengthening contributions.

The calculated contributions of each mechanism are shown in Figure 12. The theoretical values exhibit strong agreement with experimental results, confirming the validity of the strengthening model.

### 4.2. Toughening Mechanism

**Figure 13:**
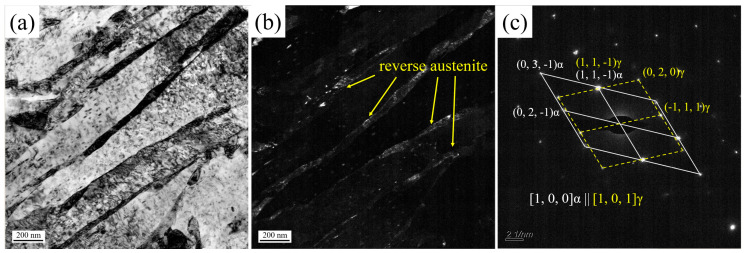
(**a**) Bright field, (**b**) central dark field using FCC diffraction spot as the operating reflection, (**c**) diffraction pattern of SQA sample.

Compared with the DQA sample, the SQA sample exhibits ~110 MPa reduction in yield strength but a ~70% improvement in impact toughness. Previous studies suggest that thin-film or nanoscale austenite often cannot be detected by XRD or EBSD [35,36]. To resolve this, TEM characterization was performed on the SQA sample (Figure 13). The morphology and distribution of austenite were identified using central dark-field imaging based on FCC diffraction spots. Central dark-field imaging revealed thin film-like reversed austenite, uniformly distributed between martensitic laths.

**Figure 14:**
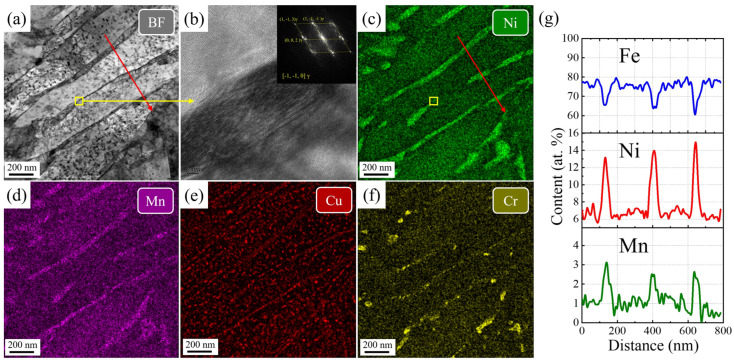
The TEM-EDS mapping in the SQA sample. (**a**) bright field image, (**b**) HRTEM image, (**c**–**f**) element mapping, (**g**) concentration profiles of the martensitic laths.

**Figure 15:**
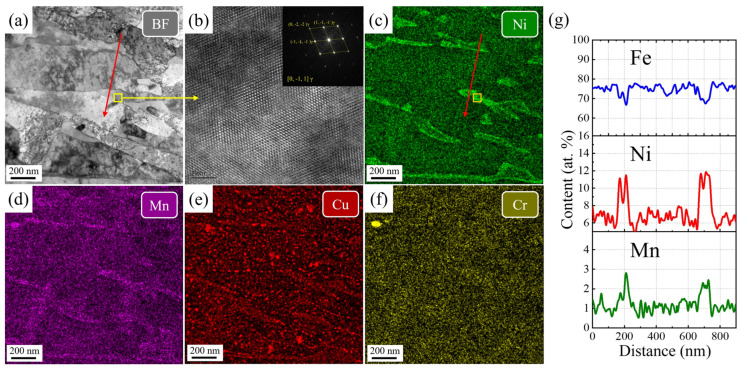
The TEM-EDS mapping in the DQA sample. (**a**) bright field image, (**b**) HRTEM image, (**c**–**f**) element mapping, (**g**) concentration profiles of the martensitic laths.

To further elucidate the toughening mechanism in the SQA and DQA sample, low-magnification EDS analysis was conducted on the martensitic laths of SQA and DQA samples, as shown in Figure 14 and Figure 15, respectively.

Figure 14 reveals the enrichment of Ni and Mn in the film-like austenite, which has a thickness of approximately 30~50 nm in the SQA sample. The Ni content in the core region ranges from 13~15 at.%, while the Mn content is about 2.5~3.5 at.%. The high-resolution image in Figure 14b further confirms that these element-enriched regions correspond to reversed austenite. In addition, segregation of Cr and the precipitation of Cr-rich carbides are observed along the lath boundaries, consistent with the findings in Figure 10. The reversed austenite formed during tempering is typically metastable and can transform into martensitic under low-temperature or stress conditions, a phenomenon commonly referred to as metastable austenite [37].

Extensive studies have demonstrated that such metastable austenite contributes to the toughening of martensitic steels, thereby effectively enhancing the toughness of high-strength steels [38]. The toughening mechanisms can be attributed to several factors. First, metastable austenite is a soft and ductile phase. When a propagating crack encounters it during fracture, the austenite undergoes plastic deformation, alleviating stress concentration at the crack tip and absorbing energy. This process results in crack blunting or deflection, thereby improving toughness [39]. Second, the deformation of metastable austenite near the crack tip induces stress relaxation in localized stress concentration zone, which delays crack initiation and impedes propagation, thus increasing the energy required for fracture [40]. Third, under high stress, metastable austenite can transform into martensite through deformation-induced transformation (the TRIP effect), which absorbs additional energy and further enhances fracture toughness [41]. Finally, during tempering, elements such as C, Mn, and Ni become enriched in reversed austenite, lowering the Ms temperature below room temperature and improving thermal stability. This enrichment also purifies the martensitic matrix by sequestering impurities, further contributing to improved toughness [42].

As shown in Figure 15, reversed austenite is also observed in the DQA sample. Although its proportion is not significantly different from that in the SQA sample, it primarily exhibits a blocky morphology, with widths exceeding 100 nm and lengths up to 500 nm. The Ni content in this reversed austenite is 10~12 at.%, while the Mn content is about 2.0~3.0 at.%, both lower than those in the SQA sample. Consequently, the toughening effect of this blocky reversed austenite is inferior to that of the film-like reversed austenite in the SQA sample. The effectiveness of metastable austenite in enhancing toughness largely depends on the TRIP effect [43]. Austenite with higher stability maximizes this effect, and its stability is influenced by two main factors: chemical composition (e.g., C, Mn, Ni) and austenite size. Higher alloying content enhances stability by lowering the Ms temperature, while finer austenite grains also improve stability [44]. In contrast, the blocky metastable austenite in the DQA sample, owing to its larger size and lower alloy element content, exhibits reduced stability and therefore provides less toughening compared with the SQA sample.

The formation of blocky metastable austenite in the DQA sample can be attributed to two main reasons. First, the as-rolled microstructure of the DQA sample contains numerous fine grains and martensitic phases, with both prior austenite grains and martensite laths being relatively disordered and exhibiting fewer parallel lath bundles [45]. Second, because the DQA sample does not undergo solution treatment, the distribution of alloying elements remains inhomogeneous [39]. As a result, during aging, alloying elements are unable to sufficiently segregate to lath boundaries.

In contrast, as shown in Figure 10, significant segregation of Mo elements at the lath boundaries is observed in the SQA sample. Mo segregation enhances the interfacial cohesion between adjacent laths, thereby inhibiting crack initiation and propagation, and improving the low-temperature toughness [46,47]. Furthermore, the SQA sample exhibits lower dislocation density and reduced stress concentration, both of which further contribute to its superior toughness [48].

## 5. Conclusions

This study systematically compared direct quenching plus aging (DQA) with solution treatment plus aging (SQA) in Cu-precipitation-strengthened steel. The main findings are as follows:

(1) A new high-strength low-carbon steel has been developed, achieving a yield strength of 1205 MPa, yield ratio of 0.93, V-notch impact toughness of 104 J at −20 °C, and elongation of 14%.
(2) DQA yields higher strength and greater work hardening. This enhancement arises from the preservation of fine recrystallized grains formed during rolling, which reduces the effective grain size. Moreover, the high dislocation density and deformation substructures provide abundant nucleation sites for Cu precipitates, leading to a higher number density and finer precipitates. The lower yield ratio is primarily attributed to the high initial dislocation density and deformation substructures, which increase work-hardening capacity and consequently reduce the yield ratio.
(3) SQA provides superior impact toughness. This improvement results from the homogeneous redistribution of alloying elements after solution treatment, which promotes the formation of regularly arranged lath structures and thin, film-like metastable reversed austenite. The presence of this austenite significantly enhances toughness through multiple toughening mechanisms. Additionally, Mo segregation at lath boundaries strengthens interfacial cohesion, further contributing to improved low-temperature toughness.

## Figures and Tables

**Table 1:** Chemical composition of the steel (wt.%).

C	Mn	Si	Cu	Ni	Cr	Mo	Nb	Ti	Fe
0.048	1.0	0.34	3.6	8.2	1.6	0.5	0.06	0.03	bal.

**Table 2:** Mechanical properties of the experimental steel.

Sample	Yield Strength (MPa)	Tensile Strength (MPa)	Yield Ratio	Elongation (%)	KV_2_ −20 °C (J)
DQ	958 ± 6	1216 ± 8	0.79	13.5 ± 0.5	134 ± 12
DQA	1205 ± 7	1291 ± 9	0.93	14 ± 0.5	105 ± 9
SQ	876 ± 3	1125 ± 8	0.78	15 ± 1	148 ± 15
SQA	1093 ± 12	1104 ± 9	0.99	13 ± 0.5	178 ± 8

## Data Availability

The data supporting the findings of this study are available within the article. Additional raw data are available from the corresponding author upon reasonable request.
